# Distinct Myocardial Transcriptomic Profiles of Cardiomyopathies Stratified by the Mutant Genes

**DOI:** 10.3390/genes11121430

**Published:** 2020-11-28

**Authors:** Katharina Sielemann, Zaher Elbeck, Anna Gärtner, Andreas Brodehl, Caroline Stanasiuk, Henrik Fox, Lech Paluszkiewicz, Jens Tiesmeier, Stefan Wlost, Jan Gummert, Stefan P. Albaum, Janik Sielemann, Ralph Knöll, Hendrik Milting

**Affiliations:** 1 Erich and Hanna Klessmann Institute, Clinic for Thoracic and Cardiovascular Surgery, Heart and Diabetes Centre NRW, Georgstrasse 11, D-32545 Bad Oeynhausen, Germany; kfrey@cebitec.uni-bielefeld.de (K.S.); AGaertner@hdz-nrw.de (A.G.); abrodehl@hdz-nrw.de (A.B.); cstanasiuk@hdz-nrw.de (C.S.); hfox@hdz-nrw.de (H.F.); lpaluszkiewicz@hdz-nrw.de (L.P.); jens.tiesmeier@muehlenkreiskliniken.de (J.T.); SWlost@hdz-nrw.de (S.W.); jgummert@hdz-nrw.de (J.G.); 2 Center for Biotechnology (CeBiTec), Bielefeld University, 33615 Bielefeld, Germany; alu@cebitec.uni-bielefeld.de (S.P.A.); janik.sielemann@uni-bielefeld.de (J.S.); 3 Graduate School DILS, Bielefeld Institute for Bioinformatics Infrastructure (BIBI), Bielefeld University, 33615 Bielefeld, Germany; 4 Department of Medicine, Integrated Cardio Metabolic Centre (ICMC), Heart and Vascular Theme, Karolinska Institutet, 141 57 Huddinge, Sweden; zaher.elbeck@ki.se; 5 Bioscience, Cardiovascular, Renal & Metabolism, BioPharmaceuticals R&D, AstraZeneca, 431 83 Gothenburg, Sweden

**Keywords:** cardiomyopathy, lamin A/C, RNA binding motif protein 20, titin, plakophilin 2, DCM, ARVC

## Abstract

Cardiovascular diseases are the number one cause of morbidity and mortality worldwide, but the underlying molecular mechanisms remain not well understood. Cardiomyopathies are primary diseases of the heart muscle and contribute to high rates of heart failure and sudden cardiac deaths. Here, we distinguished four different genetic cardiomyopathies based on gene expression signatures. In this study, RNA-Sequencing was used to identify gene expression signatures in myocardial tissue of cardiomyopathy patients in comparison to non-failing human hearts. Therefore, expression differences between patients with specific affected genes, namely *LMNA* (lamin A/C), *RBM20* (RNA binding motif protein 20), *TTN* (titin) and *PKP2* (plakophilin 2) were investigated. We identified genotype-specific differences in regulated pathways, Gene Ontology (GO) terms as well as gene groups like secreted or regulatory proteins and potential candidate drug targets revealing specific molecular pathomechanisms for the four subtypes of genetic cardiomyopathies. Some regulated pathways are common between patients with mutations in *RBM20* and *TTN* as the splice factor RBM20 targets amongst other genes *TTN*, leading to a similar response on pathway level, even though many differentially expressed genes (DEGs) still differ between both sample types. The myocardium of patients with mutations in *LMNA* is widely associated with upregulated genes/pathways involved in immune response, whereas mutations in *PKP2* lead to a downregulation of genes of the extracellular matrix. Our results contribute to further understanding of the underlying molecular pathomechanisms aiming for novel and better treatment of genetic cardiomyopathies.

## 1. Introduction

Cardiomyopathies are linked to heart failure and sudden cardiac death [1] and are traditionally classified due to their clinical phenotype into hypertrophic, dilated, restrictive, arrhythmogenic right ventricular cardiomyopathy as well as left ventricular noncompaction [2]. Genetic forms are found in all types of cardiomyopathies and genetic variants are identified in a high number of different genes which might be related to different disease courses [3]. For each of these different cardiomyopathies, penetrance, age of disease onset, survival or prevalence of arrhythmias might be clinically remarkably heterogeneous due to differences in their genetic aetiology [4,5]. Interestingly, there is a genetic overlap between different cardiomyopathies [6]. Since next generation sequencing is frequently used in cardiovascular genetic diagnostics, specific associated genotypes and mutations might be also used for a more sophisticated disease classification in clinical settings. The genetic analysis of cardiomyopathies is therefore suggested by the American Heart Association (AHA) [7], the Heart Failure Society of America (HFSA) [2] as well as the European Society of Cardiology (ESC) [8].

Non-ischemic cardiomyopathies are the most common indication for heart transplantation (HTx) [9]. However, their aetiologies are heterogeneous and genetic variants are found e.g., in proteins of the nuclear envelope, the Ca^2+^ transient, the sarcomere and the sarcolemma or the cytoskeleton [3,10]. The most frequent variants associated with dilated cardiomyopathy (DCM) are truncating mutations in the gene *TTN* (*TTNtv*) which comprise 10–25% of DCM cases [11]. *TTNtv* are associated with incomplete penetrance and late onset heart failure, whereas patients with i.e., pathogenic *RBM20* or *LMNA* mutations are affected by comparably early cardiomyopathies with incomplete penetrance [3]. Arrhythmogenic right ventricular cardiomyopathy (ARVC) is most frequently associated with defects in the desmosome [10]. The right ventricle is mainly affected, but with ongoing disease, the left ventricle is significantly affected as well [12]. Left ventricular forms of arrhythmogenic cardiomyopathy (ACM), which is a broader description of ARVC, are almost missing in the 2010 update diagnostic criteria, which are therefore not entirely specific for the disease [13,14]. However, due to advances in the clinical, pathological and genetic architecture of ARVC [14], left ventricular involvement and left-dominant or biventricular subtypes are now increasingly recognized. Further, genotype–phenotype correlation studies have recently identified clinical forms of ARVC associated with early dominant left ventricular involvement, which may parallel or even exceed the severity of right ventricular involvement [13,15,16]. The introduction of the term ACM was therefore recently proposed by the Heart Rhythm Society/European Heart Rhythm Association (HRS/EHRA) [17] to embrace the different pathologies of the disease [14]. However, there is still a lack of diagnostic and prognostic data and of the genetic basis and pathogenesis of ACM [18]. In our study, we therefore included ACM cases and compared this cardiomyopathy to other forms based on their genotypes. Mutations in the desmosomal genes cause ARVC but other genes like e.g., *FLNC* [19] can be affected as well [3]. However, the most frequent affected gene is *PKP2* (11–51%) [5] encoding the desmosomal plaque protein plakophilin 2. This protein mediates the molecular connection of the desmosomal cadherins with the cytolinker protein desmoplakin.

As several forms of cardiomyopathies show a phenotypic overlap, like left ventricular involvement in DCM as well as in two thirds of ARVC cases [20], the differentiation between these diseases is complex [1] although clinically useful [3]. However, here we detect distinct myocardial transcriptomic profiles in patients affected with different genetic cardiomyopathies in refractory heart failure (stage D) stratified by the affected genes (*LMNA*, *PKP2*, *RBM20* and *TTN*) and not based on the clinical phenotype (DCM or ARVC).

The data might be additionally useful for the identification of candidate drug targets addressing specific genetic cardiomyopathies. The aim of this study was to investigate if different genetic subtypes of DCM and ARVC are associated with specific transcriptome profiles to allow better diagnostics and deeper understanding of the molecular disease background.

The study proves that the molecular signature of the myocardium might contribute to an advanced subclassification of different monogenic cardiomyopathies based on the mutant gene.

## 2. Materials and Methods

### 2.1. Patient Cohorts

Twenty-five patients diagnosed with DCM or ARVC were included when a pathogenic or likely pathogenic variant (class 4 or 5) was detected according to the guidelines of the American College of Medical Genetics and Genomics (ACMG) [21] in only one of the following genes: *LMNA*, *RBM20*, *TTN* or *PKP2*. These genes were selected for this study, as mutations in these specific genes occur with high frequencies among cardiomyopathy patients. In total, 174 cardiomyopathy associated genes (File S2) were screened for variants in all patients using a homogeneous multigene panel to exclude potential samples with multiple pathogenic or likely pathogenic variants (class 4 or 5) according to the ACMG guidelines. Established criteria for clinical diagnostics of DCM and ARVC were used [22,23,24,25]. ARVC patients were listed due to the reduced cardiac index. Therefore, left ventricular myocardial tissue was dissected for all investigated samples. Clinical data of the patients are available in Table 1 and File S1A,B. All samples with *TTN* variants included in this study lead to a premature termination codon (*TTN*tv). In our cohort, the percentage of male patients in cardiomyopathy/non-failing (NF) groups is comparable (72% versus 67%), even though the sex-distribution of transplanted patients in general is remarkably different between males and females (about 80% of transplanted patients are males). The samples were therefore matched as well as possible according to their age. Due to the limited availability of human left-ventricular myocardial tissues, the distribution among the sample groups is heterogeneous. As proven by single factor ANOVA, there was no significant age difference between the *PKP2*, *LMNA*, *RBM20* and *TTN* groups, respectively. For comparison of the cardiac function of different patients, it is mainly focused on the cardiac index, as this value incorporates the body surface area. The cardiac index is used to normalise the cardiac output of different patients and thus makes the heart function comparable [26]. This kind of comparability is not ensured for the other parameters (left ventricular ejection fraction (LV-EF), left ventricular end-diastolic diameter (LVEDD), left ventricular end-systolic diameter (LVESD) and fractional shortening (FS)) as these are ‘independent’ from the body size. Even though LV-EF, LVEDD, LVESD and FS are different among the sample groups, the cardiac index is not.

Non-failing rejected human donor hearts were used as control samples. Donor hearts were rejected due to technical reasons and samples were only selected for RNA-Seq analysis after genotyping using the multigene panel described above. All patients were of European origin. The study was approved by the local ethics committee (No. 2018-330). All patients gave their written informed consent.

### 2.2. Sample Preparation and Sequencing Reaction

Left-ventricular myocardial tissue was directly dissected after HTx and was snap-frozen in liquid nitrogen and stored at −80 °C. RNA was isolated from about 30 mg of heart tissue using the Qiagen RNeasy Mini Kit (Hilden, Germany) according to the manufacturer’s protocol (# 74104). The RNA integrity number was measured using the Agilent RNA 6000 Pico Kit (# 5067–1513) (Santa Clara, CA, USA) and was at least above 7.8.

RNA samples were processed with the Illumina TruSeq Stranded Total RNA Library Prep Kit (San Diego, CA, USA) with Ribo-Zero Gold (# RS-122-2301) following the manufacturer’s recommendations. Final indexed libraries were enriched with seven PCR cycles. The indexed libraries were quantified with a Qubit dsDNA HS assay kit (# Q32854) (Massachusetts, MA, USA) and qualified with Bioanalyzer using an HS DNA Kit (# 5067–4627) (Santa Clara, CA, USA). Equimolar amounts of each library were pooled together and sequenced on Illumina HiSeq 3000 (single-end; 50 bp) using sequencing-by-synthesis chemistry v4 according to the manufacturer’s protocols. Each TruSeq RNA library (https://www.illumina.com/products/by-type/sequencing-kits/library-prep-kits/truseq-stranded-total-rna.html) produced an average yield of 500 Mb of sequencing data with an average of 96% of the reads achieving a quality score equal to or greater than Q30.

### 2.3. Read Processing

Mutscan (v1.14.0) [27] was used to validate the variants in the fastq files. Raw reads were quality trimmed and the remaining adapter sequences were removed using Trimmomatic (v0.27) [28]. Leading and trailing bases below a quality threshold of 3 were removed, an average read quality of 15 in a window of 4 bases was ensured and reads with less than 36 bases were discarded. The quality of the reads was examined further using FastQC (v0.11.7) [29]. Next, the RNA-Seq reads were mapped to the hg38 (GRCh38) reference genome sequence using STAR (v2.5.1b) [30]. Output filtering was determined by the parameters ‘–outFilterMismatchNoverLmax’ (filtering if the ratio of mismatches to mapped length is higher than this value) and ‘–outFilterMatchNminOverLread’ (filtering if the number of matched bases normalised to the read length is lower than this value). FeatureCounts (v1.6.4) was used to quantify annotated genes in the GRCh38.gtf file [31].

### 2.4. Data Normalisation, Principal Component Analysis and Determination of Differentially Expressed Genes

Prior to differential expression analysis, low-abundance genes were filtered out according to established methods [32]: Based on a histogram of log-mean counts for all genes, only genes with a mean count greater than 4 (log_10_(4) ≈ 0.6) in at least one condition were considered for further analysis (File S1C,D). For genes below this cutoff, it is difficult to distinguish real expression from measurement errors and sequencing noise.

The R package DESeq2 (v1.22.2) [33] was used for downstream analysis. A variance stabilising transformation was conducted. Furthermore, a principal component analysis (PCA) was performed using prcomp (stats-package (v3.5.2)) and plotted using ggplot2 (v3.1.0) [34] to investigate the distribution of the samples and the quality of the replicates. Genes which contribute most to the variance between the samples have most impact on the distribution of the points in a PCA. The read counts were normalised using the counts slot.

The DeSeq2 package (BMC Genome Biology, London, UK) allows the extraction of lists of DEGs. The parameters (a) adjusted *p*-value and (b) log2FC (for the investigation of up- and downregulation) were used to detect DEGs which are unique for one of the specific sample types associated with mutations in *LMNA*, *PKP2*, *RBM20* and *TTN*, respectively. These two parameters are part of all DEG lists relevant for the analyses performed in this study. In the manuscript text, we always specify the value of the parameter used for the specific analyses. The lists of DEGs in comparison to the control samples with an adjusted *p*-value (Benjamini–Hochberg) lower than 0.05 were obtained from the DESeq2 pipeline. One of the main goals of our study is to determine DEGs which are specific for the condition. This means that DEGs listed in our study are typically regulated in one specific subgroup. The design of our study allows the differentiation between DEGs which are truly unique for a specific genetic condition. Thus, DEGs listed for a specific genetic disease are (a) differentially regulated versus controls (non-failings) and (b) simultaneously not associated with other genetic conditions. Genes which are comparably high/low expressed in non-failing hearts and one specific genetic condition, were excluded. The inclusion of control samples is therefore crucial to identify condition specific DEGs. Specific DEGs were extracted for the four different genotypes. We defined ‘condition specific DEGs’ as those genes which are differentially expressed only in one specific genotype in comparison to non-failing samples. In other words, those DEGs are not found when other genotypes were compared to non-failing samples. For example, specific DEGs for *LMNA* samples were extracted when these genes were identified as DEGs in *LMNA* samples, but not in *PKP2*, *TTN* and *RBM20* samples. Venn diagrams were created using the R package VennDiagram (v1.6.0) [35].

### 2.5. K-Means Clustering

K-means clustering and cluster validation was performed using Clust (v1.10.7) (BMC Genome Biology, London, UK), which identifies co-expressed or correlated groups of genes [36]. Normalised counts were used as input. Within the Clust algorithm, quantile normalisation (code 101) and z-score normalisation (subtract the mean of the row and then divide by its standard deviation) (code 4) were performed as suggested for RNA-Seq data.

### 2.6. GO Term and Pathway Analysis

Regulated Gene Ontology (GO) terms and pathways were identified using the R package gage [37]. The analysis was performed using a publicly available workflow [38]. The bioinformatic analysis performed here was done to identify genotype specific GO terms. The non-failing controls were introduced as a reference used to compare each sample type against all other samples. DESeq2 was used for statistical analysis. The filtered read counts (Section 2.4) were used as input for these analyses. For example, *LMNA* samples were compared directly against *PKP2*, *RBM20*, *TTN* and control samples in one single step using DESeq2. This means that the samples were divided into two groups (e.g., LMNA versus (*PKP2* + *RBM20* + *TTN* + control samples)) for downstream analysis with DESeq2 prior to the actual pathway and GO term extraction [38].

GO terms were obtained from the list ‘go.sets.hs’ as provided by the gageData package (BMC Genome Biology, London, UK). Fold changes, calculated with DESeq2, were used as input for the gage function. For pathway analysis the DESeq function was applied to the dataset for differential expression analysis. AnnotationDbi (v1.44.0) [39] and org.Hs.eg.db (v.3.7.0) [40] were applied for annotation of human genes and associated pathways. The obtained results were visualised using the R package and function pathview [41] as well as the Python package plotly [42].

### 2.7. Prediction of Secreted Proteins, Regulatory Proteins and Potential Candidate Drug Targets

Lists of transcription factors and predicted secreted proteins were obtained from the human protein atlas. Using the lists of specific unique DEGs of all genetic conditions, extracted as described in Section 2.4, regulated transcription factors as well as secreted proteins were identified based on the overlap of the lists of DEGs with the lists from the protein atlas. The threshold for mean expression of control samples was set to lower than or equal to three as secreted proteins should ideally only be expressed in perturbed metabolic states when used as disease specific markers. Further, a list of druggable genes was obtained from Finan et al. [43]. Specific DEGs for each genotype separately (*LMNA*, *PKP2*, *RBM20* and *TTN*) (*p*adj < 0.01) were defined as potential disease modifying genes. The overlap between the list of druggable genes, which are a subsection of all human protein coding genes, and the list of potential disease modifying genes [44] was used to predict potential candidate drug targets which are specific and unique for each of the investigated sample types.

## 3. Results

In total, 25 patients with genetic cardiomyopathies caused by mutations (class 4 or 5 according to the ACMG guidelines) in four different genes were investigated to identify transcriptomic differences. Six patients carry pathogenic mutations in *LMNA*, in six additional patients *PKP2* is affected, truncating mutations in *TTN* were found in nine patients and *RBM20* is affected in four patients (File S1B). All patients with mutations in *PKP2* were clinically diagnosed with ARVC according to the revised task force criteria [25], whereas all other patients with mutations in one of the other three genes were initially diagnosed with DCM. These four genes were selected for this study as mutations in *LMNA*, *PKP2*, *TTN* and *RBM20* occur with high frequencies among cardiomyopathy patients. Six samples obtained from non-failing human hearts were used as control samples. For the analyses performed in the study, we focused on unique and specific DEGs, pathways, GO terms and predicted secretory/regulatory proteins and potential drug targets, which only occur in samples with one affected gene, but not in samples with all other affected genes. This allows the comparison of molecular differences based on the affected, mutant gene and not based on the stage of heart failure or the clinical phenotype (DCM/ARVC).

**Figure 1:**
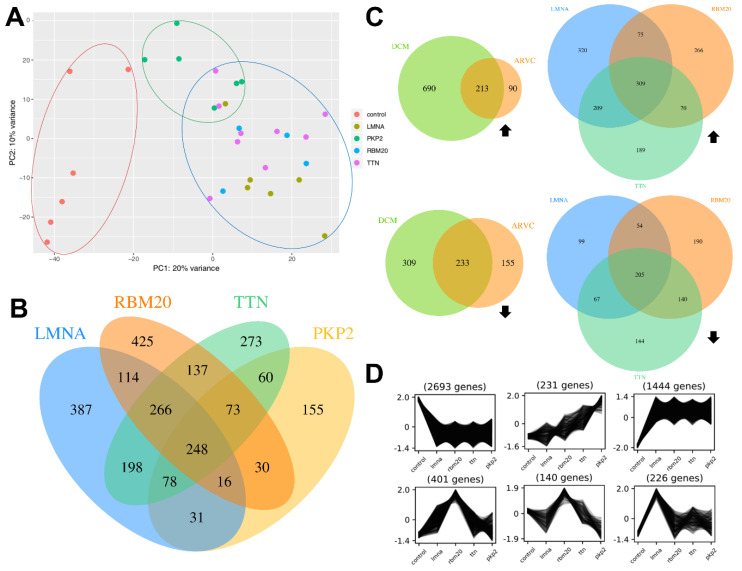
Data overview. (**A**) Principal component analysis (PCA) of all samples. The different classes (I) control, (II) dilated cardiomyopathy (DCM) and (III) arrhythmogenic right ventricular cardiomyopathy (ARVC) are marked with red, blue and green circles, respectively. (**B**) Venn diagram displaying the amount of DEGs (padj < 0.05, |log2FC| > 1) of all genotypes in comparison to the control samples. (**C**) Venn diagrams displaying upregulated (top) and downregulated (bottom) DEGs (padj < 0.05, |log2FC| > 1) of DCM samples and ARVC samples in comparison to the control samples (**left**) as well as of *LMNA*, *RBM20* and *TTN* associated samples in comparison to control samples (**right**). Black arrows indicate up-/downregulation. (**D**) K-means clustering of the expression data of all sample types (x-axis) using the normalised counts as input. The y-axis represents the normalised expression values. The expression patterns show groups of genes which are co-expressed and therefore specifically regulated for one of the five sample types.

The PCA provides a first overview regarding the distribution of the samples. The first component, in which the samples are separated, explains 20% of the total variance (extracted by gene level). The separation of DCM, ARVC and control samples is shown and marked with blue, green and red circles, respectively (Figure 1A). No global difference was observed for samples with mutations in *LMNA*, *RBM20* and *TTN*. A similar pattern can be observed in the PCA excluding *PKP2* samples (File S1F). Control samples cluster separately from samples with mutations in *LMNA*, *RBM20* and *TTN* with PC1 explaining 24% of the total variance.

Further, DEGs for DCM and ARVC were identified (Figure 1C). In summary, 446 genes are significantly differentially expressed (padj < 0.05, |log2FC| > 1) in both sample types. DCM is associated with 999 specific DEGs and 245 genes are specifically differentially expressed in ARVC samples of which more genes are upregulated in DCM samples (690 upregulated, 309 downregulated), whereas more downregulated (155) than upregulated (90) DEGs were identified for ARVC samples.

Analysis of DEGs for each sample type as well as k-means clustering revealed substantial differences between all genotypes. Several genes are specifically up- or downregulated in one of the sample types as shown in the Venn diagram (Figure 1B,C; File S2) as well as the cluster profiles (Figure 1D). For all sample types, specific DEGs were detected. Samples with pathogenic variants in *LMNA*, *RBM20*, *TTN* and *PKP2* have 248 DEGs in common. Figure 1D shows six characteristic cluster profiles obtained after analysis with Clust using the normalised counts as input. In total, 13 different cluster profiles were generated (File S1E) which revealed genes with different expression patterns according to the respective genotype (File S2). The first cluster profile (top left) contains 2693 genes, which are co-expressed and all highly expressed in control samples compared to all other genotypes. 226 genes are exclusively upregulated in the myocardium of laminopathy patients in comparison to the other analysed conditions (Figure 1D; bottom, right).

### 3.1. GO Term and Pathway Analysis

A classification of all DEGs was achieved using the Gene Ontology. A large number of GO terms were significantly (*p* < 0.05) different in each sample type compared to all other investigated sample types. For samples with mutations in *LMNA*, *PKP2*, *RBM20* and *TTN*, 542, 585, 652 and 1107 significantly different GO terms (upregulated + downregulated) were identified, respectively. The tables in File S2 including regulated GO terms contain upregulated as well as downregulated GO terms for each genetic condition.

Samples with pathogenic variants in *LMNA* are widely associated with an upregulation of genes involved in the immune system (File S2). ‘Lymphocyte activation’, ‘immune response-regulating cell surface receptor signaling pathway’ and ‘T cell activation’ are the top three upregulated GO terms, whereas genes of the mitochondrial membrane are significantly downregulated. These data might indicate an aseptic inflammatory response involved in laminopathies as recently suggested by Brayson et al. [45]. Genes associated with the mitochondrial matrix are upregulated in samples with mutations in *PKP2* (File S2). Regarding this genotype, the extracellular matrix (ECM) is affected as the corresponding genes are significantly downregulated. Mutations in *RBM20* lead to an upregulation of genes involved in muscle development and muscle contraction (File S2). A downregulation of genes involved in immune response is visible for samples with mutations in *RBM20* as well as for samples with mutations in *TTN* (File S2). Further, one of the significantly upregulated GO terms regarding this sample type is calcium activated cation channel activity.

Most importantly, we demonstrate, that regulated pathways differ depending on the mutant gene although the clinical phenotype is shared (File S2).

Several differentially regulated pathways were detected between all investigated sample types. For subjects with mutations in *LMNA*, 39 (18 upregulated, 21 downregulated) significantly (*p* < 0.05) regulated pathways were detected. Regarding samples with pathogenic variants in *PKP2*, 21 (14 upregulated, 7 downregulated) pathways were significantly differentially regulated compared to 35 (5 upregulated, 30 downregulated) significantly differentially regulated pathways in samples with mutations in *RBM20*. The myocardium of subjects with titinopathies is associated with 50 (0 upregulated, 50 downregulated) significantly regulated pathways compared to the other investigated sample types, including the control.

**Figure 2:**
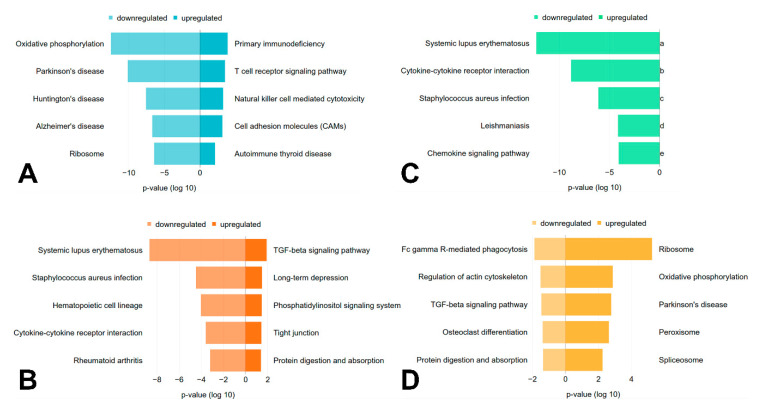
Regulated pathways. The top five significantly (*p* < 0.05, parametric two-sample *t*-test) up- and downregulated pathways for each genotype (**A**) *LMNA*, (**B**) *RBM20*, (**C**) *TTN*, (**D**) *PKP2* in comparison to all other sample types are shown, if available. The x-axis represents the *p*-value (log 10), which is ‘negative’ for downregulated pathways and ‘positive’ for upregulated pathways.

For samples with mutations in *LMNA*, immune pathways are upregulated, whereas the most significantly downregulated pathway is ‘oxidative phosphorylation’ (Figure 2A). Three of the five most significantly downregulated pathways for the samples with variants in *RBM20* and *TTN* are shared (Figure 2B,C). The TGFβ signaling pathway is significantly upregulated in samples with mutations in *RBM20* (Figure 2B) and significantly downregulated in samples with mutated *PKP2* (Figure 2D). Furthermore, some pathways, like specific infection or disease pathways, are significantly regulated.

### 3.2. Prediction of Secreted Proteins, Regulatory Proteins and Potential Candidate Drug Targets

Further characterisation of DEGs was achieved by classification into secretory proteins, regulatory proteins/transcription factors and potential drug targets (File S2). For each genotype, specific unique DEGs were used to predict potential proteins of each class. This analysis was performed to detect potential disease markers (secretory proteins), as well as proteins which could play a key role in the regulation of disease specific mechanisms (regulatory proteins) based on the affected gene and not on the clinical phenotype.

Table 2 provides an overview regarding potential regulatory proteins as well as potential secretory proteins. The adjusted *p*-value as well as the respective log2FC of predicted proteins of each class are shown.

Potential candidate drug targets were identified by bioinformatic prediction. In total, 133, 86, 128 and 56 potential drug targets were bioinformatically predicted for the samples with mutations in *LMNA*, *RBM20*, *TTN* and *PKP2*, respectively.

## 4. Discussion

In total, 25 patients with different monogenic cardiomyopathies caused by mutations in four distinct genes were investigated to identify transcriptomic differences.

We analysed in this project terminal failing hearts from two different cardiological diseases/phenotypes. The clinical data were derived from the listing protocol for heart transplantation. All myocardial samples were derived from terminal failing patients with refractory heart failure requiring transplant or ventricular assist device (VAD)-implantation, respectively. We included two different forms of cardiomyopathies, ARVC and DCM, which might have influence on the gene expression profiling. However, the primary goal of this study was to evaluate the myocardial gene expression profiling in human myocardium which is affected by ACMG class 4 or 5 mutations.

Although the cardiac indices of patients with ARVC or DCM in this cohort are not significantly different there might be an influence of the right ventricular function (see differences for the parameters LV-EF, LVEDD, LVESD and FS (Table 1)) on the cardiac index and the left ventricular gene expression profile. However, genomics data comparing DCM versus controls and ARVC myocardium from heart transplantation candidates was published from our group before [46]. In that paper, the data were stratified by the clinical phenotypes of the HTx-candidates. A clear separation of failing and non-failing samples was found but not for DCMs and ARVCs. In that study the molecular genetics of ARVCs were heterogeneous and those of the DCM patients unknown. Thus, the genetic aetiology of the ARVC- and DCM-cohorts in that study was heterogeneous as well. In addition, it is known from the genetic background of cardiomyopathies that the affected gene is not directly related to the cardiac disease phenotype suggesting that there is a phenotypic overlap of different monogenic cardiomyopathies [6]. For example, *PKP2* mutations in general were reported before to be associated with ACM, NCCM and DCM-phenotypes. Consequently, we and others found that a *PKP2*-missense mutation might be of relevance for the development of a DCM as well [47]. In addition, the pathogenic heterozygous genotype *PLN* p.R14del might be associated with DCM or ARVC even in closely related family members for unknown reasons [48]. Thus, the identical genotype might therefore be associated with the development of remarkably different cardiomyopathy phenotypes.

For these reasons we hypothesized that the stratification by the genotype will reveal more precisely different affected pathways as compared to the clinical phenotype in the failing human myocardium. Consequently, we stratified in this study by the patient’s genotype and proved the clustering accordingly. Therefore, the inclusion criteria of our study were based primarily on the monogenic genotype of non-ischemic cardiomyopathies and not on their cardiac phenotype. We integrated in our manuscript for these reasons left ventricular samples of two different cardiomyopathies, which, however, can also be classified by their genetic aetiology.

The PCA reveals a clear separation of controls and samples with variants in *PKP2*, which are associated with ARVC. A clearer separation of the data might be achieved by elimination of variance within the dataset e.g., by investigating the myocardium of patients with the same age, same gender or same body mass index. However, this was not possible due to the lack of available patients with equal traits and conditions.

Nevertheless, clear differences between all four sample types were detectable when analysing DEGs. Each sample type is associated with its specific set of DEGs indicating specific molecular expression signatures for different genetic cardiomyopathies depending on the mutant gene. This is also detectable for sample types with the same clinical phenotype (DCM) (Figure 1C). Specific and unique DEGs were identified for samples with mutations in *LMNA*, *RBM20* and *TTN*, separately, even though the disease classification (DCM) is equal.

The highest number of DEGs was determined for samples with pathogenic mutations in *RBM20*. This leads to the assumption that the myocardium of these patients reacts with a strong response when exposed to the dysfunctional spliceosomal RNA binding protein. As *RBM20* is a splice factor, mutations in this gene have an early impact on other genes on RNA level and therefore might trigger a regulatory cascade affecting many downstream proteins.

K-means clustering revealed characteristic expression patterns for many genes regarding all investigated conditions. Different expression patterns indicate a distinct regulation of gene expression based on the affected gene (*LMNA*, *RBM20*, *TTN*, *PKP2*) and not only due to the disease classification (DCM versus ARVC). The investigation of translational regulation would further contribute to the understanding of the correlation between affected gene and specific underlying molecular mechanisms.

### 4.1. GO Term and Pathway Analysis

To classify the DEGs into functional categories, regulated GO terms were assigned to each sample type. Samples with pathogenic variants in *LMNA* are widely associated with an upregulation of genes involved in the immune system. These findings were reported previously as mutations in lamins trigger cytokine production and immune response [49]. Genes of the mitochondrial membrane are significantly downregulated in samples with mutations in *LMNA* and significantly upregulated in subjects with pathogenic variants in *PKP2*. Dysfunctions of the mitochondrial electron transport chain were previously linked to heart failure [50]. An increase in electron transport chain activity might be an energetic compensatory mechanism, whereas a decrease in activity might occur to protect the myocardium from overload and might result in an energy deficit and contractile dysfunction [51]. A differentiation between genotypes allows a closer investigation of electron transport chain activities and their effect on cardiac function. Regarding samples with mutations in *PKP2*, the extracellular matrix is affected as the corresponding genes are significantly downregulated. Disordered extracellular domains result in lower affinity of desmosomal cadherins [52] and furthermore might diminish force generation and mechanical resistance in the myocardium. This could contribute to loosening of cell contacts and death of cardiomyocytes [52]. In addition, rare mutations in genes involved in the coupling of the cardiomyocytes to the ECM like *FLNC* or *ILK* have been recently described in ARVC families [53,54]. As desmosomes are additionally important to maintain the coordination of cardiomyocytes [55], an affected ECM and therefore lower affinity of desmosomal cadherins might contribute to ventricular arrhythmia.

Mutations in *TTN* and *RBM20* lead to an upregulation of genes associated with calcium activated cation channel activity and transporter and channel activities, respectively. Fluctuation of intracellular calcium modulates contractility in cardiomyocytes [56] which could have an detrimental impact on cardiac function.

In summary, GO term analysis revealed substantial differences according to the respective genotype as several specific groups of genes are significantly up- or downregulated.

The regulated pathways for subjects with laminopathies are consistent with the affiliated regulated GO terms. Immune pathways are upregulated [49], whereas the most significant downregulated pathway (oxidative phosphorylation) is associated with the mitochondrial membrane (Figure 2A). Three of the five most significant downregulated pathways for the samples with mutations in *RBM20* and *TTN* are shared between these sample types (Figure 2B,C). This observation could be the result of alternative splicing as the splice factor *RBM20* targets, amongst other genes, *TTN*. The mutation of *TTN* on DNA level as well as its incorrect splicing due to the mutated splice factor *RBM20* could result in a similar cellular response on pathway level. However, *RBM20* related cardiomyopathies reveal a more severe clinical course when compared to titinopathies with e.g., earlier onset and severe disease expression [57]. A possible explanation might be the expression of multiple misspliced cardiac genes in the myocardium of *RBM20* patients that have cooperative damaging effects. Functional analysis of myocardial samples obtained from patients with *TTN*tv has previously been performed, but apart from significantly decreased stiffness, no titin related functional defects have been observed [58]. In addition, *TTN*tv have also been inserted by clustered regularly interspaced short palindromic repeats (CRISPR) in embryonic stem cell derived cardiomyocytes revealing effects on force generation [59].

For subjects with titinopathies, several significantly downregulated signaling pathways were identified, including e.g., the MAPK-, VEGF- and chemokine signaling pathway (File S2). In previous studies, *TTN*tv was already associated with the downregulation of major signaling pathways, like e.g., MAPK signaling, which is consistent with our findings [59,60]. The reduced activation of these signaling pathways might as well explain why we did not identify any unique, significantly upregulated pathways for samples with mutations in *TTN*.

In addition, some pathways, like specific infection or disease pathways, are significantly regulated. As immune response related genes, like immunoglobulin G or interleukin-1 are differentially regulated, pathways like ‘Systemic lupus erythematosus’, ‘*Staphylococcus aureus* infection’ or ‘Rheumatoid arthritis’ were detected. Further, pathways like Parkinson’s, Huntington’s and Alzheimer’s disease are regulated as several connexins are differentially expressed in samples associated with variants in *LMNA* and may point to possible similarities in the underlying molecular mechanisms [49,61]. The TGFβ signaling pathway is significantly upregulated in samples with mutations in *RBM20* (Figure 2B) and significantly downregulated in the myocardium of patients with mutations in *PKP2* (Figure 2D). An upregulation of this pathway related to DCM was described previously, as TGFβ is involved in cardiac remodelling [62]. Additionally, ARVC can be caused in rare cases by mutations in the promoter region of *TGFβ3* [63]. However, due to multiple, often opposing effects of TGFβ signaling, its molecular understanding is complex and its exact role in heart disease is still incompletely understood [62]. As a different regulation pattern of the TGFβ signaling pathway was observed in samples with mutations in *RBM20* and *PKP2*, this pathway might play a distinct role in different forms of cardiomyopathies.

Depending on the mutant gene, regulated pathways differ, even though the disease classification is shared. This underlines the relevance to differentiate cardiomyopathies also depending on their genetic aetiology to investigate their specific molecular pathomechanisms.

### 4.2. Prediction of Secreted Proteins, Regulatory Proteins and Potential Candidate Drug Targets

Further characterisation of DEGs was achieved by classification into secretory proteins, regulatory proteins/transcription factors and potential candidate drug targets by bioinformatic prediction. The detected transcription factors could play a key role in the regulation of genotype specific mechanisms and the predicted secretory proteins might be used as candidates for genotype specific biomarkers. However, to examine these possible functions further knowledge on the role of these proteins on molecular level as well as additional experimental investigations are needed.

The prediction of potential drug targets is the first important step for drug discovery in academia as well as for the pharmaceutical development and can speed up the discovery process substantially [64]. The druggable genes which were used as input for the prediction consist of targets of approved small compounds, biotherapeutic drugs and clinical-phase drug candidates. They contain also genes encoding targets with known bioactive drug-like small molecule binding partners as well as with ≥50% identity with approved drug targets and genes encoding secreted or extracellular proteins, proteins with more distant similarity to approved drug targets and members of key druggable gene families [43]. This comprehensible high-quality list of druggable genes and the following prediction of potential drug targets unique for the investigated genotype-specific heart disease might provide the first step for a specific drug discovery and the development of a personalised therapy of cardiomyopathies. In total, 129, 125, 128 and 56 potential unique drug targets were bioinformatically predicted for the genotypes *LMNA*, *RBM20*, *TTN* and *PKP2*, respectively. However, due to the limitations of bioinformatic in silico prediction, a closer investigation and verification of those targets is of course required.

### 4.3. Limitations of the Study

There are several factors which might influence differential analysis. Some limitations must be considered due to the heterogeneity of the study population (File S1A). Although, we used panel sequencing of the 174 most likely cardiomyopathy associated genes, it might be possible that further rare variants in other genetic regions are present. We also analysed a limited number of samples. A larger number of cases might improve the reliability of the results. Moreover, due to the relatively limited availability of human left-ventricular myocardial tissue, the age distribution among the sample groups is heterogeneous. The samples could also not be matched by gender as about 80% of the transplanted patients in general are males (compare database of the International Society for Heart and Lung Transplantation (ISHLT)). This gender difference as well as the limited availability of human tissues are the reasons why e.g., no females could be included in the *RBM20* sample group. An impact of age and gender differences on the gene expression pattern cannot be excluded.

We stratified the patients included in this study by their genotype. However, we analysed patients with different forms of non-ischemic cardiomyopathies. We cannot exclude that hemodynamic differences between ARVC and DCM patients might also contribute to the gene expression profiling. However, we observed significant molecular differences based on the affected gene within one single monogenic cardiomyopathy (DCM). This reveals that the incorporation of genetic information might be crucial to distinguish different cardiomyopathies and might contribute to the clinical (phenotypic) classification of the disease.

Tissue samples of the explanted hearts were consistently taken from the same region in the left ventricular free walls of HTx patients and NF hearts using standard operation procedures. However, myocardial tissue samples from LVAD implantation were from the LV-apex for technical reasons. Further, *RBM20* cases are rare and might be in general younger when compared i.e., to *TTN*tv-genotypes. Therefore, *RBM20* cases tend to be younger than others, even though the samples selected from our biobank were matched as best as possible (according to the availability of human tissues) by age. However, in our cohort the age was not significantly different.

## 5. Conclusions

Disease-causing mutations appear in a heterogeneous group of genes which can all result in cardiomyopathies. Despite the genetic heterogeneity, previous studies tried to find a common property or pattern for a particular disease type. Bowles N.E., Bowles K.R. and Towbin proposed the ‘final common pathway’ hypothesis [65]. Since variants in genes coding for specific groups of sarcomeric proteins, are associated with specific diseases, like hypertrophic cardiomyopathy, this disease is sometimes referred to as ‘sarcomyopathy’. However, as several mutations in sarcomeric proteins can also cause different forms of cardiomyopathies, the ‘final common pathway’ hypothesis does not seem sufficient to fully explain the aetiology of cardiomyopathies. Moreover, others claim that the common pathway is only used to justify common treatments and neglects underlying molecular pathomechanisms [66].

Until now, cardiomyopathies were mainly classified according to their clinical appearance (phenotype). Here, we took the first step to distinguish cardiomyopathies based on the affected genes. In our RNA-Seq analyses we revealed substantial differences in regulated GO terms, pathways and further specific genes coding for regulatory proteins, secretory proteins or potential candidate drug targets between the investigated disease subtypes, respectively. Genes of calcium activated channels as well as some significantly regulated pathways are common between patients with mutations in *RBM20* and *TTN* as the splice factor *RBM20* targets, amongst other genes, *TTN* leading to a similar response on pathway level. The myocardium of patients with mutations in *LMNA* is widely associated with upregulated genes and pathways involved in immune response, like cytokine production, whereas mutations in *PKP2* lead to a downregulation of genes of the ECM, which might diminish force generation and lead to lower affinity of desmosomal cadherins resulting in ventricular arrhythmia.

There are significant molecular differences in the myocardial transcriptomes based on the mutant gene, even though the clinical phenotype (type of cardiomyopathy) seems identical: For example, DCM patients with mutations in *LMNA* show unique, significantly differentially regulated genes, pathways and GO terms which were not identified in the myocardial transcriptome of samples with mutations in *RBM20*, even though clinically, both sample types are derived from patients with DCM. Our analysis shows that a classification of cardiomyopathies into subtypes based on the mutant genes is essential for deeper understanding of the specific cellular pathomechanisms and to finally develop personalised medicine driven therapies.

With this study, major insights into distinct molecular mechanisms of specific subtypes of cardiomyopathy based on the mutant genes have been revealed that contribute to an all-encompassing understanding of the underlying cellular effects of cardiomyopathies and which might result in improved treatment of this serious disease in the future.

## Figures and Tables

**Table 1:** Comparison of clinical findings of all sample types.

	*LMNA* (*N* = 6)	*RBM20* (*N* = 4)	*TTN* (*N* = 9)	*PKP2* (*N* = 6)	Significance (*p*-Value; ANOVA)
age	51 ± 10	38 ± 14	50 ± 15	53 ± 13	0.3037 *
gender (m:f)	2:4	4:0	7:2	5:1	-
CI (L/min/m^2^)	1.66 ± 0.46	2.00 ± 0.41	2.27 ± 0.56	1.85 ± 0.49	0.1663 *
LV-EF (%)	23.3 ± 4.7	26.5 ± 10.5	23.9 ± 4.3	52.5 ± 15.4	2.4 × 10^−5^ *
LVEDD (mm)	60.5 ± 3.6	71.5 ± 7.0	68.3 ± 6.8	46 ± 9.7	1.9 × 10^−5^ *
LVESD (mm)	52.7 ± 4.3	64.8 ± 6.3	61 ± 9.5	34.5 ± 13.0	7.5 × 10^−5^ *
FS (%)	12.7 ± 3.6	9.3 ± 3.9	8.3 ± 2.3	26.3 ± 11.6	2.4 × 10^−4^ *
disease	DCM	DCM	DCM	ARVC ^1^	-
treatment (HTx:LVAD-IP)	6:0	3:1	8:1	6:0	-
ICD	6/6	4/4	6/9	4/6	-
CAD	0	1/4	0	0	-

Abbreviations: ARVC = arrhythmogenic right ventricular cardiomyopathy, CAD = coronary artery disease, CI = cardiac index, DCM = dilated cardiomyopathy f = female, FS = fractional shortening, HTx = heart transplantation, ICD = implantable cardioverter-defibrillator, LVEDD = left ventricular end-diastolic diameter, LVESD = left ventricular end-systolic diameter, LV-EF = left ventricular ejection fraction, LVAD = left ventricular assist device, m = male, ^1^ task force criteria. ± indicates the standard deviation (mean ± s.d.), * single factor ANOVAs (F-test) were calculated to determine if the means are equal between all sample types.

**Table 2:** Predicted regulatory and secreted proteins. Genotype-specific DEGs were used for identification of regulatory proteins (padj < 0.01, |log2FC| > 1) and predicted secreted proteins (padj < 0.05). Lists of transcription factors and predicted secreted proteins were obtained from the human protein atlas. Potential regulatory proteins are shown on the left and secreted proteins on the right.

Sample Type	Predicted Regulatory Proteins			Predicted Secretory Proteins		
	Gene Name	padj	log2FC	Gene Name	padj	log2FC
*LMNA*	*ZNF117* *NKX2-5* *FOXO1* *RUNX2* *TBX3* *BCL11B* *ZFHX4* *IKZF3* *ZFHX3* *LEF1* *NHLH2* *TFCP2L1* *ONECUT2* *RORC* *ZNF165* *EOMES*	5.02 × 10^−11^ 1.78 × 10^−8^ 2.77 × 10^−6^ 9.06 × 10^−6^ 0.000258 0.000397 0.000619 0.000736 0.001378 0.002262 0.004392 0.004813 0.005433 0.005826 0.007406 0.009681	1.103 −1.069 1.172 1.631 −1.028 2.578 1.370 2.257 1.053 1.628 4.256 1.439 2.020 −1.136 −1.197 2.535	*MYL1* *OSTN* *LEP* *NPFFR2* *HTRA4* *GRIA2* *DPEP2* *CNDP1* *COL20A1* *PIANP*	0.000208 0.000791 0.000820 0.002066 0.004334 0.008678 0.022995 0.026027 0.036507 0.041499	2.982 3.258 5.106 2.671 2.028 2.616 1.925 2.002 2.196 2.189
*PKP2*	*HHEX* *MEOX1* *HLF* *BCL3* *ARID5A* *FOXS1* *HOPX* *PITX3*	0.000275 0.000871 0.000889 0.000923 0.002452 0.005327 0.006332 0.006866	−1.556 −1.464 1.142 −1.168 −1.087 −1.440 −3.044 1.960	*GABRG3*	0.043429	2.024
*RBM20*	*ZNF827* *PITX2* *TSC22D2* *ALX4* *IRF7* *SOX30* *BACH2* *MYC* *NR2F1* *CEBPD* *GATA5*	8.12 × 10^−8^ 1.53 × 10^−5^ 3.95 × 10^−5^ 0.000567 0.000808 0.002415 0.002869 0.004139 0.004371 0.005437 0.007913	1.127 5.766 1.002 −1.949 −1.040 1.353 1.023 −1.138 3.039 −1.041 2.265	*WFDC3*	0.038394	2.028
*TTN*	*ZNF676* *SREBF1* *FOXK1* *TCF7L1* *HESX1* *POU6F2* *MYF6* *BHLHE40* *BHLHE22*	5.47 × 10^−5^ 9.02 × 10^−5^ 0.000394 0.001645 0.004402 0.007423 0.009001 0.009302 0.009983	1.0369 −1.148 1.060 1.044 1.335 1.426 −3.230 −1.137 −1.462	*UPK3B* *MUC3A*	0.012358 0.047826	2.334 1.841

## Supplementary Materials

The following are available online at https://www.mdpi.com/2073-4425/11/12/1430/s1, File S1: Clinical data and supplementary figures, File S2: Supplementary Data.
