# Supplementary material for: Distinct Myocardial Transcriptomic Profiles of Cardiomyopathies Stratified by the Mutant Genes
Source: Genes (Basel). 2020 Nov 28;11(12):1430. doi: 10.3390/genes11121430 (PMC7768427; doi:10.3390/genes11121430)
Supplement: Supplementary file 1 [file genes-11-01430-s001.zip › genes-997621-SI/File S1.pdf]

A **Clinical data.** Clinical findings in patients with DCM and ARVC.

Sample Number	Diagnosis	Gender	Surgical Treatment (ST)	Age at ST (years)	NYHA	ICD	CI (L/min/m^2)	LVEDD (mm)	LVESD (mm)	FS (%)	LVEF (%)
1	DCM	m	LVAD [1]	21	4	+	1.74	81	71	13	39
2	DCM	m	LVAD [1]	23	3-4	-	n.a.	63	45	n.a.	26
3	ARVC	f	HTx	31	4	+	1.48	35	23	34	60
4	DCM	m	HTx	32	3	+	1.72	70	65	7	20
5	DCM	f	HTx	38	3	+	2.13	57	52	5	24
6	DCM	f	HTx	33	3-4	+	1.78	60	52	13	25
7	DCM	m	HTx	46	3-4	+	1.85	79	74	6	18
8	DCM	m	HTx	44	4	+	1.93	71	67	5	16
9	ARVC	m	HTx	51	4	+	1.68	42	32	23	56
10	ARVC	m	HTx	49	4	-	1.20	64	59	12	22
11	DCM	f	HTx	51	4	+	1.60	64	58	9	25
12	DCM	m	HTx	40	4	+	1.92	67	n.a.	9	23
13	DCM	m	HTx	56	3	+	1.55	65	58	10	29
14	DCM	f	HTx	48	3	+	1.60	60	50	17	22
15	DCM	f	HTx	58	4	+	2.44	55	49	10	24
16	DCM	m	HTx	53	3-4	+	2.6	64	56	12	31
17	ARVC	m	HTx	60	3	+	2.10	46	35	20	55
18	DCM	m	HTx	48	4	-	2.99	77	70	9	25
19	DCM	m	HTx	59	4	+	2.40	69	65	6	16
20	DCM	m	HTx	61	4	+	2.06	64	57	11	29
21	ARVC	m	HTx	65	4	+	2.09	44	24	45	65
22	DCM	m	HTx	62	4	+	1.00	59	49	17	15
23	ARVC	m	HTx	63	4	-	2.56	45	34	24	57
24	DCM	m	HTx	63	3	+	3.20	68	61	10	27
25	DCM	f	HTx	69	4	-	1.64	71	64	10	27
26	rejected donor heart	m	organ removal	28	n.a.	-	n.a.	n.a.	n.a.	n.a.	n.a.
27	rejected donor heart	f	organ removal	40	n.a.	-	n.a.	n.a.	n.a.	n.a.	n.a.
28	rejected donor heart	m	organ removal	50	n.a.	-	n.a.	n.a.	n.a.	n.a.	n.a.
29	rejected donor heart	m	organ removal	60	n.a.	-	n.a.	n.a.	n.a.	n.a.	n.a.
30	rejected donor heart	m	organ removal	61	n.a.	-	n.a.	n.a.	n.a.	n.a.	n.a.
31	rejected donor heart	f	organ removal	64	n.a.	-	n.a.	n.a.	n.a.	n.a.	n.a.

Abbreviations: CI = cardiac index, f = female, FS = fractional shortening, HTx = heart transplantation, ICD = implantable cardioverter defibrillator, LVAD = left ventricular assist device, LVEDD=left ventricular end-diastolic diameter, LVEF=left ventricular ejection fraction, LVESD = left ventricular end-systolic dimension, m = male, n.a. = not assessed, NYHA = New York Heart Association functional classification of heart failure, [1]=pre-VAD, NYHA classification and echocardiographic data were not available for rejected donor hearts as these data are not acquired before organ removal.

B Variants of DCM and ARVC patients. *RBM20*, *LMNA*, *TTN* and *PKP2* gene mutations in DCM and ARVC patients. For each investigated patient, sample number, patient id, affected gene, nucleotide change, rs number (if available), chromosome and position of the variant in the GRCh38 assembly are shown.

**Sample number**	**Kind of CM**	**Affected gene**	**Nucleotide change**	**Reference SNP number**	**Chromo-some**	**Position (GRCh38)**	**Mutation DNA change (ACMG, class4-5)**	**Mutation protein change (ACMG, class4-5)**	**ACMG**	**ACMG-Criteria**	**Minor allele frequency**	**Type**
1	DCM	*RBM20*	C>T	rs267607003	10	110812310	c.1913C>A	p.P638L	5	PS1, PS3, PS4, PM1, PM2, PP1, PP3, PP4	0	missense
2	DCM	*TTN*	A>T		2	178575127	c.71005A>T	p.K23669X	4	PVS1, PM2, PP4	0	nonsense
3	ARVC	*PKP2*	G>C	rs193922674	12	32802557	c.2146-1G>C	unknown	4	PVS1, PM2, PP4	0.00003184	Splice site mutation
4	DCM	*RBM20*	C>T	rs267607003	10	110812310	c.1913C>A	p.P638L	5	PS1, PS3, PS4, PM1, PM2, PP1, PP3, PP4	0	missense
5	DCM	*TTN*	delA		2	178741762	c.11483delA	p.N3828MfsX4	4	PVS1, PM2, PP4	0	deletion, frameshift
6	DCM	*LMNA*	delCAAGCTGG-CCCTGGACAT		1	156136059	c.1095_1112delCAAG CTGGCCCTGGACAT	p.I365_D370del	4	PM1, PM2, PM4, PP4	0	in-frame deletion
7	DCM	*TTN*	delTG		2	178633448	c.42909_42910delTG	p.C14303WfsX12	4	PVS1, PM2, PP4	0	deletion, frameshift
8	DCM	*RBM20*	T>C	rs794729154	10	110821360	c.2741T>C	p.V914A	4	PS3, PM2, PP1, PP4	0	missense
9	ARVC	*PKP2*	T>A		12	32822499	c.1939_1961del23	p.C647fsX88	4	PVS1, PM2, PP4	0.00003184	Deletion, frameshift
10	ARVC	*PKP2*	G>C	rs193922674	12	32802557	c.2146-1G>C	unknown	4	PVS1, PM2, PP4	0.00003184	Splice site mutation
11	DCM	*LMNA*	delCT	rs59684335	1	156135280-156135285	c.904_905CT	p.S303CfsX27	5	PVS1, PS3, PM2, PP4	0	deletion, frameshift
12	DCM	*TTN*	T>G		2	178533512	c.103103T>G	p.L34368X	4	PVS1, PM2, PP4	0	nonsense
13	DCM	*LMNA*	C>T	rs267607601	1	156115024	c.106C>T	p.Q36X	4	PVS1, PM2, PP4	0	nonsense
14	DCM	*LMNA*	C>T	rs59026483	1	156134457	c.568C>T	p.R190W	5	PS1, PS3, PM1, PM2, PP3, PP4	0	missense
15	DCM	*LMNA*	C>T	rs267607554	1	156135925	c.961C>T	p.R321X	5	PVS1, PS3, PM2, PP4	0	nonsense
16	DCM	*RBM20*	C>G	rs1114167331	10	110812301	c.1904C>G	p.S635C	5	PS3, PM1, PM2, PM5, PP1, PP3, PP4	0	missense

17	ARVC	*PKP2*	C>T	rs886041322	12	32878222	c.658C>T	p.Q220X	4	PVS1, PM2, PP4	0	Nonsense
18	DCM	*TTN*	C>T	rs72646828	2	178598904	c.56806C>T	p.R18936X	4	PVS1, PM2, PP4	0.000004037	nonsense
19	DCM	*TTN*	G>A		2	178573100	c.73032G>A	p.W24344X	4	PVS1, PM2, PP4	0	nonsense
20	DCM	*TTN*	C>G	rs1114167324	2	178590043	c.61682C>G	p.S20561X	4	PVS1, PM2, PP4	0	nonsense
21	ARVC	*PKP2*	delCT	rs794729129	12	32824048	c.1803delC	p.D601EfsX655	4	PVS1, PM2, PP4	0.00003185	Deletion, frameshift
22	DCM	*LMNA*	G>A	rs28933093	1	156130741	c.481G>A	p.E161K	5	PS1, PS3, PM1, PM2, PP3, PP4	0	missense
23	ARVC	*PKP2*	C>T	rs1325285497	12	32802526	c.2176C>T	p.Q726X	4	PVS1, PM2, PP4	0	Nonsense
24	DCM	*TTN*	A>T		2	178575127	c.71005A>T	p.Lys23669Ter	4	PVS1, PM2, PP4	0	nonsense
25	DCM	*TTN*	insA	rs1440081449	2	178581636-178581637	c.66632dupA	p.N22211KfsX8	4	PVS1, PM2, PP4	0	insertion, frameshift

Minor allele frequency according to the Genome Aggregation Database (gnomAD, https://gnomad.broadinstitute.org/), 2020-04-03.
Used NM- or NP-numbers are NM_001134363.3 or NP_001127835.2 for *RBM20*, NM_170707.4 or NP_733821.1 for *LMNA*, NM_001267550.1 or NP_001254479.1 for *TTN* and NM_001005242.3 or NP_004563.2 for *PKP2*.

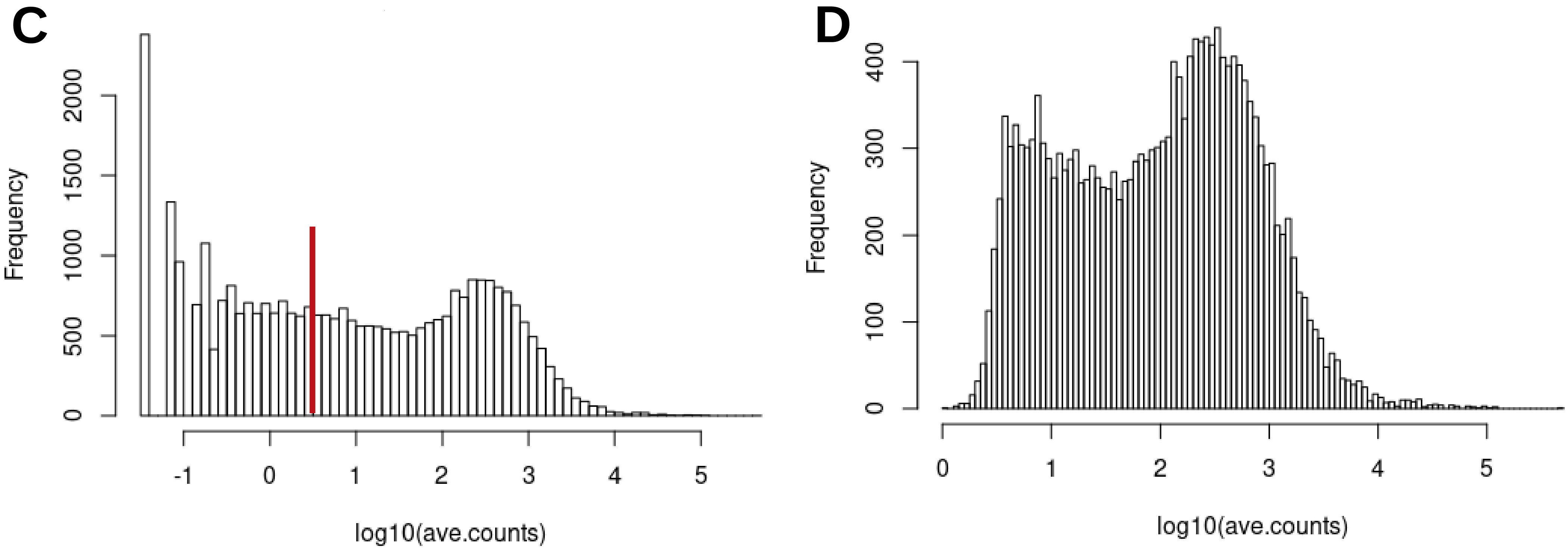

Figure S1, C+D: Histogram of log-mean counts for all genes before (C) and after (D) the cutoff (red line). Only genes with a mean count greater than 4 ($\log_{10}(4) \approx 0.6$) in at least one condition were considered for further analysis. For genes below this cutoff it is difficult to distinguish real expression from measurement errors and sequencing noise.

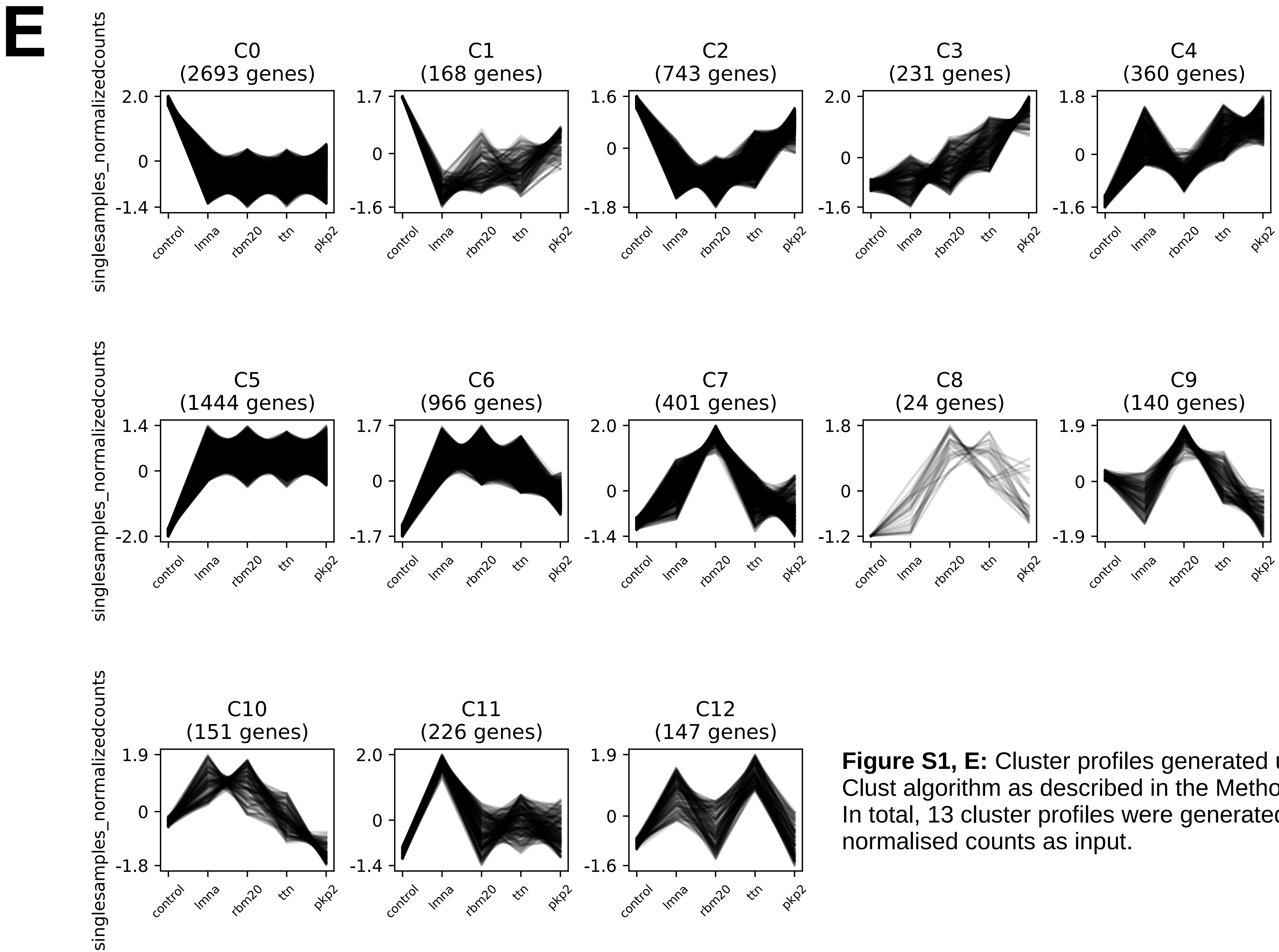

Figure S1, E: Cluster profiles generated using the Clust algorithm as described in the Method section. In total, 13 cluster profiles were generated using the normalised counts as input.

F

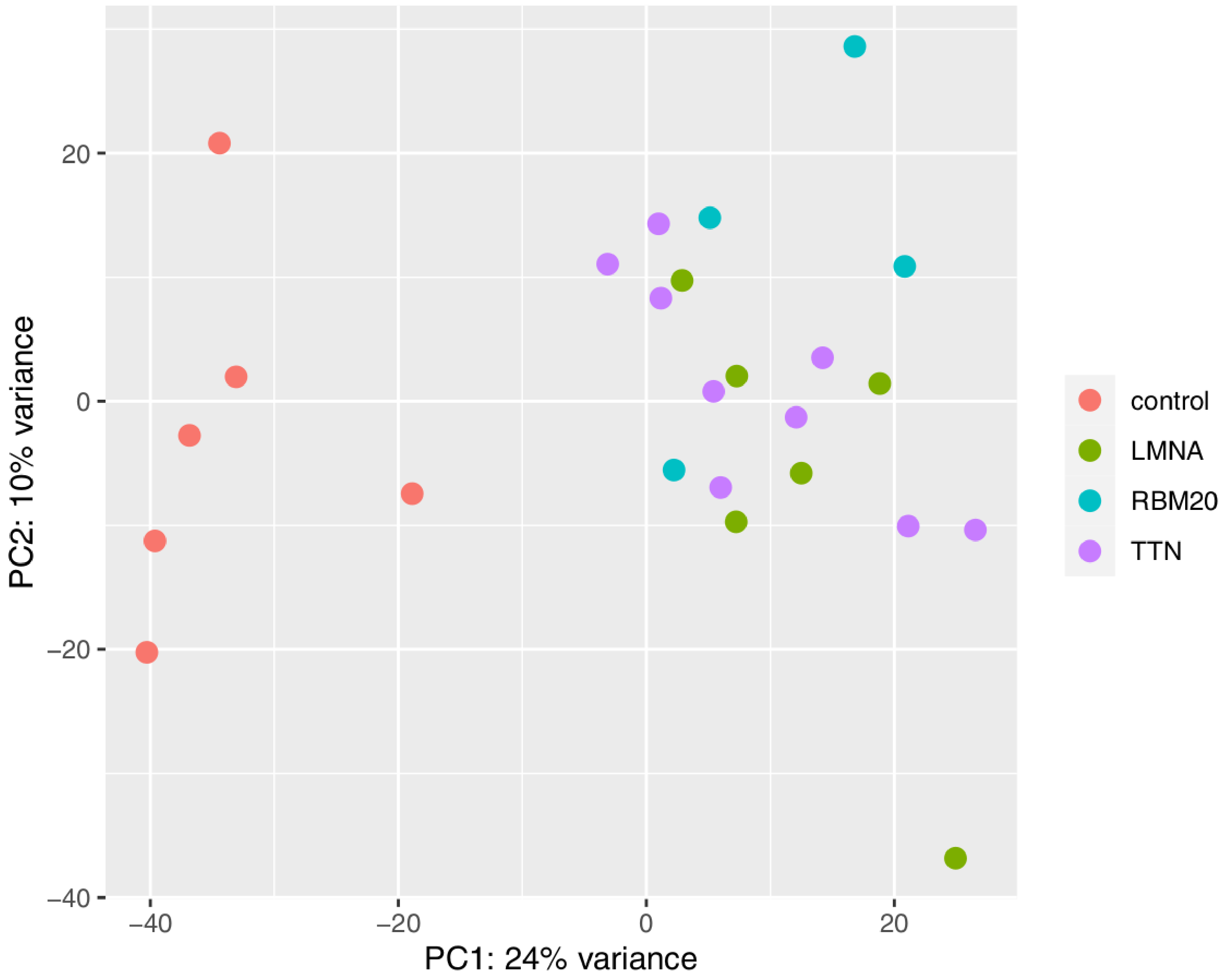

Figure S1, F: Principal component analysis excluding *PKP2* samples. In comparison to Figure 1A, a similar pattern can be observed, with PC1 explaining 24% of the total variance.
